# Developing a single strain for in vitro salvage synthesis of NAD^+^ at high temperatures and its potential for bioconversion

**DOI:** 10.1186/s12934-019-1125-x

**Published:** 2019-04-25

**Authors:** Hironori Taniguchi, Makoto Imura, Kenji Okano, Kohsuke Honda

**Affiliations:** 1 Department of Biotechnology, Graduate School of Engineering, Osaka University, Yamadaoka 2-1, Suita, Osaka 565-0871 Japan; 2 KOHJIN Life Sciences Co., Ltd., Higashihama 1-6, Saiki, Oita, 876-858 Japan

**Keywords:** NAD^+^, In vitro metabolic engineering, Thermophilic enzyme, Artificial operon, Cascade reaction, OGAB

## Abstract

**Background:**

Thermostable enzymes have several advantages over their mesophilic counterparts for industrial applications. However, trade-offs such as thermal instability of enzyme substrates or co-factors exist. Nicotinamide adenine dinucleotide (NAD^+^) is an important co-factor in many enzyme-catalyzed oxidation–reduction reactions. This compound spontaneously decomposes at elevated temperatures and basic pH, a property that limits catalysis of NAD^+^/NADH-dependent bioconversions using thermostable enzymes to short timeframes. To address this issue, an “in vitro metabolic pathway” for salvage synthesis of NAD^+^ using six thermophilic enzymes was constructed to resynthesize NAD^+^ from its thermal decomposition products at high temperatures.

**Results:**

An integrated strain, *E. coli* DH5α (pBR-CI857, pGETS118-NAD^+^), that codes for six thermophilic enzymes in a single operon was constructed. Gene-expression levels of these enzymes in the strain were modulated by their sequential order in the operon. An enzyme solution containing these enzymes was prepared by the heat purification from the cell lysate of the integrated strain, and used as an enzyme cocktail for salvage synthesis of NAD^+^. The salvage activity for synthesis of NAD^+^ from its thermal decomposition products was found to be 0.137 ± 0.006 µmol min^−1^ g^−1^ wet cells. More than 50% of this initial activity remained after 24 h at 60 °C. The enzyme cocktail could maintain a NAD^+^ concentration of 1 mM for 12 h at 60 °C. Furthermore, this enzyme cocktail supported continuous NAD^+^/NADH-dependent redox reactions using only NAD^+^/NADH derived from host cells, without the need for addition of external NAD^+^.

**Conclusions:**

The integrated strain allows preparation of an enzyme cocktail that can solve the problem of NAD^+^ instability at high temperatures. The strain simplifies preparation of the enzyme cocktail, and thus expands the applicability of the in vitro metabolic engineering method using thermostable enzymes. Further optimization of gene expressions in the integrated strain can be achieved by using various types of ribosome binding sites as well as promoters.

**Electronic supplementary material:**

The online version of this article (10.1186/s12934-019-1125-x) contains supplementary material, which is available to authorized users.

## Background

Enzymes with high thermal stability have great potential for industrial high-temperature applications [1, 2]. Reactions at high temperatures have several advantages such as low risk of contamination by microorganisms, decreased liquid viscosity, increased solubility of substrates and high mass transfer rates [3]. High structural rigidity allows thermostable enzymes to retain activity at high temperatures and in extreme conditions such as in organic solvents [4–6].

Recombinant thermostable enzymes can be expressed in mesophilic host cells, and simple heat incubation can eliminate undesired mesophilic enzyme activity of the host organism, while retaining desirable thermostable enzyme activity. This approach eliminates the need for complicated protein purification steps, and increases utility of thermostable enzymes in small scale reactions and in locations where special facilities are not available. Exploiting these advantages, thermostable enzymes have been integrated into in vitro enzymatic cascade reactions, a process often referred as in vitro metabolic engineering [7]. Using enzymes from thermophiles, several studies have reported production of lactic acid [8], malic acid [9], butanol [10], hydrogen [11], and *myo*-inositol [12, 13] from carbohydrates.

An obstacle to the use of thermostable enzymes is thermal instability of nicotinamide adenine dinucleotide, NAD^+^/NADH [9–11]. NAD^+^/NADH is an essential co-factor for many enzyme-catalyzed redox reactions, but both NAD^+^ and NADH are thermolabile [14, 15]. This reduces the efficiency and reaction timeframes of in vitro metabolic engineering at high temperatures [9, 16].

**Fig. 1:**
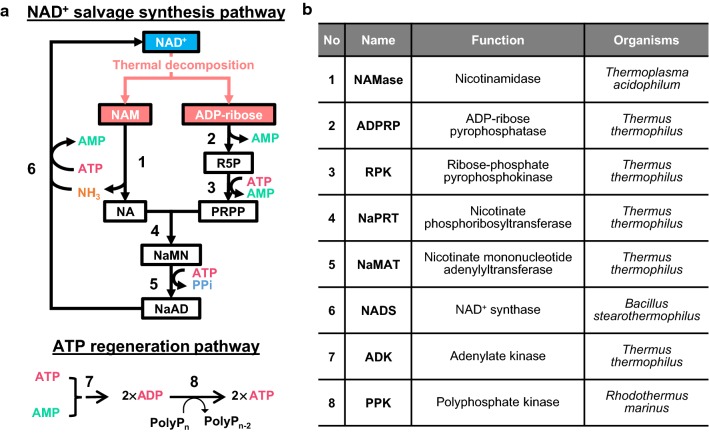
In vitro NAD^+^ salvage synthesis pathway. **a** In vitro pathway for NAD^+^ salvage synthesis (upper scheme) and for the ATP regeneration (lower scheme) are illustrated. Chemical substances are shown in the box (NAM, nicotinamide; NA, nicotinic acid; R5P, ribose-5-phosphate; PRPP, phosphoribosyl pyrophosphate; NaMN, nicotinic acid mononucleotide; NaAD, nicotinic acid adenine dinucleotide). **b** Gene name, function, and source of organism are summarized. The number of the gene is corresponding to the number in **a**

NAD^+^ is more stable at acidic pH; NADH is more stable at basic pH [14]. Organisms including thermophilic organisms possess a metabolic pathway to resynthesize NAD^+^ from its thermal decomposition products, nicotinamide (NAM) and ADP-ribose [17]. This pathway inspired construction of an in vitro metabolic pathway to resynthesize NAD^+^ from its thermal decomposition products. The pathway was optimized for use at a basic pH of 8, wherein NADH degradation is suppressed (Fig. 1a) [18]. The pathway includes six thermophilic enzymes for NAD^+^ salvage and two thermophilic enzymes for regeneration of AMP to ATP, and was able to maintain NAD^+^ concentration at 60 °C for several hours.

Thermal instability of nicotinamide cofactors is a general obstacle for in vitro metabolic engineering at high temperatures. In vitro reconstitution of NAD^+^ salvage pathway using individually prepared thermophilic enzymes enables us fine tuning of the loading ratio of each enzyme (i.e., the optimum distribution of a given total amount of enzymes) to maximize the rate of NAD^+^ salvage through the in vitro pathway [18]. On the other hand, a burden lies on cultivation of multiple strains expressing different enzymes and preparation of an enzyme cocktail at a proper ratio upon each use, particularly at a large-scale bioconversion. These procedures hinder reaping the benefit of thermophilic enzymes. A single integrated strain that expresses all six thermophilic enzymes necessary for NAD^+^ salvage synthesis would allow users to omit those laborious steps, and facilitates the use of thermostable enzymes for redox reaction at high temperatures. In this study, such a strain was constructed, and the enzyme cocktail prepared from the resulting strain was shown to support enzymatic redox reactions at high temperatures. Construction of this integrated strain and associated enzyme cocktail expands the repertoire of reactions that can be effectively catalyzed using thermophilic enzymes.

## Results

In vitro adjustment of enzyme activities to achieve a high metabolic flux (i.e., a high production rate of desired products) can be accomplished by changing enzyme loading in the reaction mixture. This approach is not applicable to an integrated strain in which a series of thermophilic enzymes composing a pathway are co-expressed. In such case, enzyme activity needs to be tuned at the level of in vivo expression. Such tuning of enzyme ratios was addressed at the level of mRNA [16]. Expression of genes in a single operon generally decreases as the distance between promoter and gene increases [19]. Using this concept, an artificial polycistronic operon was designed to include the six genes required for NAD^+^ salvage synthesis, so that transcription would be controlled by a single promoter.

The operon was constructed using genes described in the previous study [18]. These genes code for nicotinamidase (NAMase) from *Thermoplasma acidophilum*, ADP-ribose pyrophosphatase (ADPRP), ribose-phosphate pyrophosphokinase (RPK), nicotinate phosphoribosyltransferase (NaPRT), nicotinate mononucleotide adenylyltransferase (NaMAT) from *Thermus thermophilus*, and NAD^+^ synthase (NADS) from *Bacillus stearothermophilus* (Fig. 1b). In the current study, codon-optimized sequences of these genes were used to eliminate the bias of codon usage and translational rate on protein expression.

### Determination of gene order in the artificial operon

**Fig. 2:**
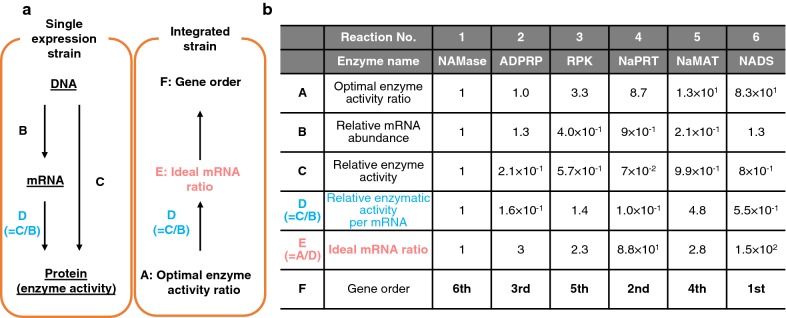
Determination of gene order in an artificial operon. **a** The scheme to calculate ideal mRNA amounts for each gene is illustrated. “Ideal mRNA ratio (E)” can be calculated from “Enzyme activity per mRNA (D)” and “Optimal enzyme activity ratio (A)”. **b** Experimentally determined values are shown. Values are normalized to that of the gene encoding NAMase

Gene order in the artificial operon was determined by ranking the ideal abundance of mRNA of each gene, which was calculated from “Enzyme activity per mRNA” and “Optimal enzyme activity ratio” (Fig. 2a). A series of single-gene-expression strains was initially constructed to individually express each of the six enzymes involved in the salvage synthesis of NAD^+^. Subsequently, abundance of mRNAs in each of these strains was determined by qRT-PCR with corresponding primer sets (Additional file 1: Figure S1). Concurrently, activity of individual enzymes was determined as described in the methods section (Additional file 1: Figure S2). Enzyme activity per mRNA was calculated from mRNA abundance and enzyme activity in each single-gene-expression strain (Fig. 2b). Optimal enzyme activity ratios for NAD^+^ salvage synthesis were determined experimentally in the previous work [18]. More precisely, enzymes prepared from each single expression strain were individually titrated without changing the concentration of other components, and the optimal ratio ideal for NAD^+^ salvage synthesis was determined [18]. Relative amounts of the mRNA ideal to balance enzyme activity were calculated, and the order of genes in the operon was identified as: 1st-NADS, 2nd-NaPRT, 3rd-ADPRP, 4th-NaMAT, 5th-RPK and 6th-NAMase.

### Strain construction and confirmation of enzyme production

**Fig. 3:**
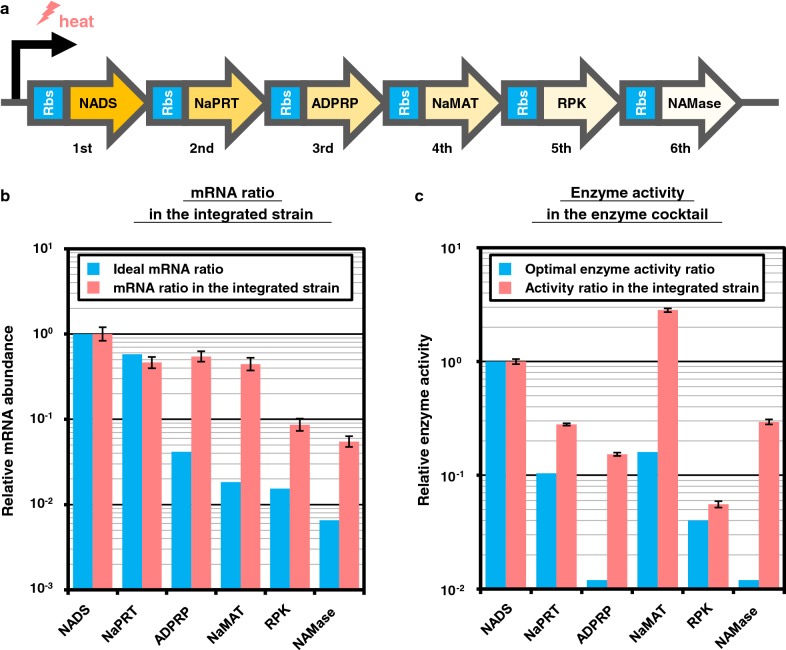
mRNA and enzyme activity ratio in the integrated strain. **a** The construct of an artificial operon is illustrated. The order of genes is determined as shown in Fig. 2b. Each gene contains an identical ribosome-binding site, and its expression is regulated by a single temperature-inducible promoter. **b** mRNA ratio of each gene in the integrated strain is shown. Blue bars indicate the calculated ideal ratio of mRNA for NAD^+^ salvage shown in Fig. 2b (line E), and, red bars indicate the ratio of mRNA measured in the integrated strain. Y axis shows the mRNA ratio in log scale, which is normalized to the expression level of 1st gene encoding NADS. **c** Enzyme activity in the heat-purified enzyme solution prepared from the integrated strain is shown. Blue bars indicate the optimal enzyme activity ratio for salvage synthesis of NAD^+^ at 60 °C shown in Fig. 2b (line A), and red bars indicate the activity of each enzyme in the integrated strain. Y axis shows enzyme activity in log scale, which is normalized to that of 1st gene encoding NADS. The error bar represents standard error calculated from triplicate measurements

A plasmid with the artificial operon was constructed using OGAB (ordered gene assembly in *Bacillus subtilis*) [20] as described in the methods section (Additional file 1: Figure S3). The resulting plasmid, pGETS118-NAD^+^ was co-transformed into *E. coli* with pBR-CI857, which encodes a temperature-sensitive repressor, cI857, and thus enables the induction of gene expression from pGETS-118-NAD^+^ upon a shift in cultivation temperature. This integrated strain, *E. coli* DH5α (pBR-CI857, pGETS118-NAD^+^), produces all six thermostable enzymes necessary for NAD^+^ salvage synthesis in a temperature-inducible manner (Fig. 3a).

Abundance of mRNA and enzyme activity of each gene in the integrated strain were measured by qRT-PCR and spectrophotometry, respectively (Fig. 3b, c). Abundance of mRNA showed decreasing trend as the distance between promoter and gene location increased. For the genes encoding ADPRP, NaMAT, RPK, NAMase, genes in the 3rd, 4th, 5th, and 6th-position, relative mRNA abundance normalized against that of the 1st gene of NADS was approximately 10 times higher than the ideal mRNA abundance calculated (Fig. 3b). Activities of all the enzymes were confirmed in the integrated strain. When enzyme activity was normalized to that of NADS encoded by the first gene in the operon, the activity of all enzyme except NADS in the enzyme cocktail (indicated as Red bars in Fig. 3c) was higher than that of optimal enzyme activity ratio determined experimentally (indicated as Blue). This result indicated that NADS is the rate-limiting enzyme in the salvage cocktail prepared from the integrated strain. Relatively high enzyme activity of NaMAT, NAMase, and ADPRP in the integrated strain was possibly caused by a high abundance of their mRNAs.

**Fig. 4:**
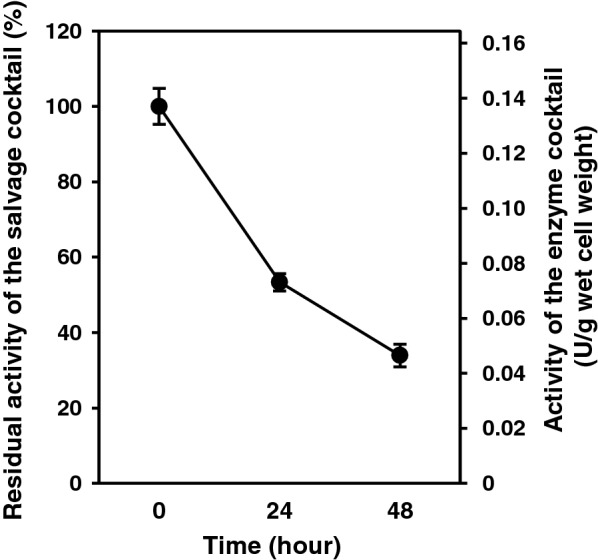
Overall performance of the enzyme cocktail and its thermal stabilities. The activity of the enzyme cocktail to synthesize NAD^+^ from NAM and ADP-ribose is shown. The activity of the salvage cocktail was measured after incubation at 60 °C for 0, 24 and 48 h. Residual activities after 24 and 48 h were normalized to the activity without incubation (0 h). The absolute activity was calculated against wet cell weight used for the preparation of enzyme cocktail. One unit is defined as the activity to produce 1 µmol of NAD^+^ per minute. The error bar represents standard error calculated from triplicate measurements

Overall performance of the enzyme cocktail from the integrated strain was assessed by coupling the salvage reaction with a thermophilic glucose dehydrogenase (GDH) using spectrophotometry (Additional file 1: Figure S2), and NAD^+^ production rate from NAM and ADP-ribose was monitored (Fig. 4). The enzyme cocktail synthesized NAD^+^ from these substrates at a rate of 0.137 ± 0.006 µmol min^−1^ g^−1^ wet cells. The cocktail as a whole maintained 53.3 ± 2.3% and 33.9 ± 4.1% of the initial activity of NAD^+^ salvage synthesis after 24 and 48 h of incubation at 60 °C, respectively (Fig. 4).

### Salvage synthesis of NAD^+^ by the enzyme cocktail

**Fig. 5:**
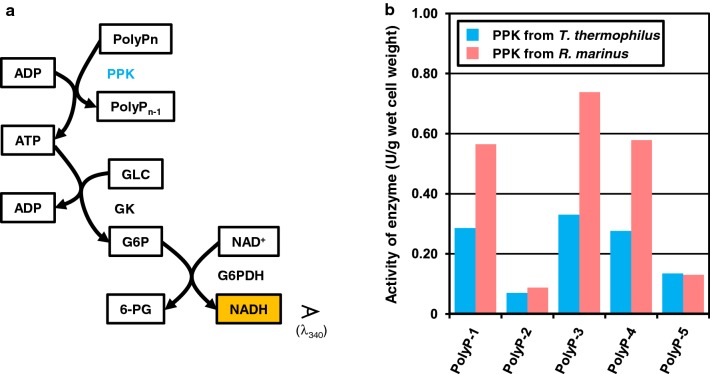
PPK screening and their polyphosphate dependency. **a** Spectrometric quantification of PPK activity is illustrated. G6P; glucose-6-phosphate, 6-PG; 6-phosphogluconic acid. **b** The activity of PPK from *T. thermophilus* and *R. marinus* are shown. Following polyphosphate were used in the assay; PolyP-1 (purchased from Nacalai Tesque, Cat: 27704-15; Japan); PolyP-2 (FUJI Film, Cat: 194-05935; Japan); PolyP-3 (Sigma, Cat: 28-2880-5; Germany); and PolyP-4 (Sigma, Cat: 1.06529; Germany). PolyP-5 was prepared as describe elsewhere [18]

Regeneration of ATP from AMP is necessary for sustainable NAD^+^ salvage synthesis. Salvaging one molecule of NAD^+^ consumes three molecules of ATP, which are converted to AMP (Fig. 1). The coupled reaction of adenylate kinase (ADK) and polyphosphate kinase (PPK) to regenerate ATP from AMP was thus integrated into salvage synthesis [21] (Fig. 1). We performed a screening of PPK from different organisms suitable for reaction conditions of salvage enzymes, and the one from *Rhodothermus marinus* was selected. The combination with PolyP-3 (Cat: 28-2880-5; Sigma, Germany) and PPK from *R. marinus* showed the highest activity (Fig. 5). This combination was used for NAD^+^ salvage synthesis in this work. Screening of ADK was not performed, because ADK from *T. thermophilus* showed sufficient activity under the experimental condition.

**Fig. 6:**
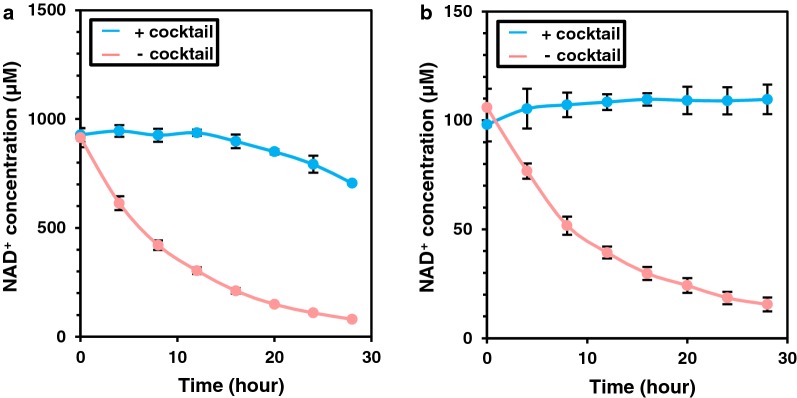
NAD^+^ salvage synthesis at 60 °C. Concentration of NAD^+^ at 60 °C was monitored every 4 h for 28 h with the enzyme cocktail (+ cocktail) or in the negative control (− cocktail). Initial concentration of NAD^+^ was 1 mM (**a**) and 100 µM (**b**). Error bars represent standard errors calculated from triplicates measurements

NAD^+^ salvage synthesis was performed with both NAD^+^ salvage synthesis cocktail and ATP regeneration enzymes at 60 °C. Total volume of enzyme solutions, which were prepared from 200 mg wet cells/mL of cell suspensions, was 22.5% (vol/vol) of the reaction mixture. As a negative-control, the same volume of a heat-treated cell lysate prepared from an *E. coli* strain that expresses no thermophilic enzymes was added instead of the NAD^+^-salvage cocktail. Starting with 1 mM of NAD^+^, NAD^+^ concentration was maintained for 12 h by the salvage cocktail (94%, 937 ± 14 μM), then gradually decreased over time (Fig. 6a). After 28 h, NAD^+^ concentration was 71% (705 ± 6 μM) of the initial concentration compared to 8% (80 ± 4 μM) in the negative control. Starting with 100 μM of NAD^+^, NAD^+^ concentration was maintained for at least 28 h by the cocktail (110%, 110 ± 7 μM), while the concentration decreased to 16 ± 3 μM (16%) in the negative control (Fig. 6b). The NAD^+^ salvage cocktail was thus capable of maintaining substantial NAD^+^ concentration at high temperatures and the duration was depending on the initial concentration of NAD^+^ as well as on the loading amount of the enzyme cocktail.

### Coupling salvage synthesis with enzyme-catalyzed redox reactions

**Fig. 7:**
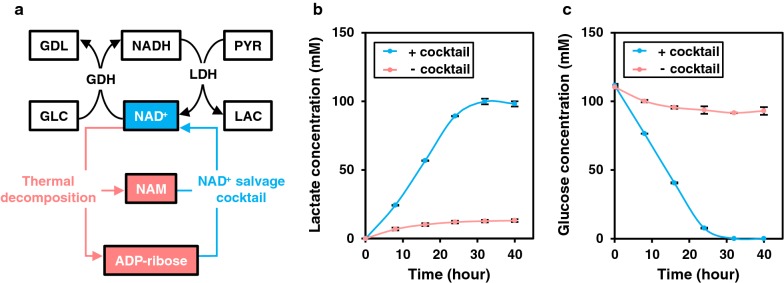
NAD^+^/NADH dependent redox reaction without exogenous NAD^+^. **a** Scheme of the NAD^+^/NADH-dependent redox reaction with the NAD^+^ salvage cocktail is shown. GDH and LDH represent glucose dehydrogenase and lactate dehydrogenase, respectively. Chemical substances are shown in the box (GLC, glucose; GDL, glucono δ-lactone; PYR, pyruvate; LAC, lactate). Lactate (**b**) and glucose (**c**) concentrations were measured every 8 h for 40 h with the enzyme cocktail (+ cocktail) or in the negative control (− cocktail). Error bars represent standard errors calculated from triplicate measurements

**Fig. 8:**
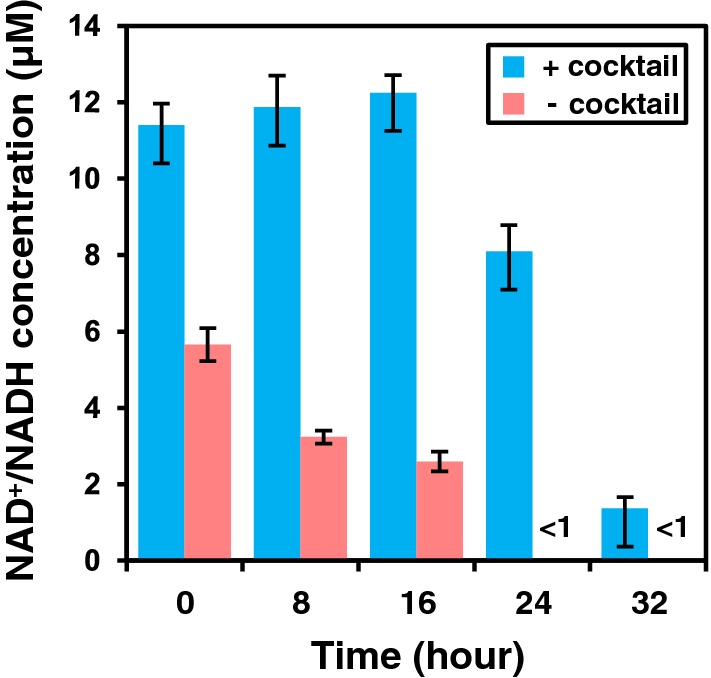
NAD^+^/NADH concentration. NAD^+^/NADH concentrations were measured every 8 h for 32 h with the enzyme cocktail (+ cocktail) or in the negative control (− cocktail). NAD^+^/NADH concentrations in the negative control were below the detection limit (< 1 µM). Error bars represent standard errors calculated from triplicate measurements

To evaluate compatibility of salvage synthesis with other thermophilic enzyme reactions, the salvage cocktail was combined with the coupled reactions of pyruvate to lactate and glucose to gluconolactone by lactate dehydrogenase (LDH) and GDH, both of which catalyze NAD^+^/NADH dependent redox reactions (Fig. 7a). Initial concentrations of pyruvate and glucose were 100 mM. The NAD^+^ salvage cocktail was replaced in negative controls with the heat-treated cell lysate from the parental strain. When 100 µM of NAD^+^ was added, a slight effect of the enzyme cocktail was noted by lactate production at 6 h (79.1 ± 0.9 mM and 69.6 ± 2.3 mM in the presence and absence of the enzyme cocktail, respectively). In contrast, complete conversion of pyruvate and glucose was achieved even in the negative control (Additional file 1: Figure S4). This result is likely due to the low *K*_m_ (~ 10 µM) of LDH and GDH for NADH and NAD^+^, respectively [22, 23]. On the other hand, when no exogenous NAD^+^ was added to the reaction mixture, complete conversion of pyruvate to lactate and glucose to gluconate was achieved only in the presence of the salvage cocktail (Fig. 7b, c). With the enzyme cocktail, glucose is consumed at a constant rate (4.3 mM h^−1^), and 99.9 ± 2.1 mM of lactate was produced after 32 h. In the negative control, glucose concentration decreased over time and plateaued after 24 h, resulting in the final lactate titer of 13.1 ± 0.5 mM. With the salvage cocktail, total concentration of NAD^+^ and NADH was initially 11.4 ± 0.6 µM at 0 h, and inclusion of the salvage cocktail maintained levels above 10 µM for at least 16 h (Fig. 8). Initial concentration of NAD^+^ and NADH (5.7 ± 0.4 µM) was lower in the negative control, and decreased over time to undetectable levels after 24 h (< 1 µM). Higher initial NAD^+^/NADH concentrations in the presence of the salvage cocktail was possibly caused due to NAD^+^ salvage synthesis activity during preparation of enzyme solutions at 70 °C for 30 min. Results indicate that NAD^+^ decomposition products generated during heat inactivation of host enzymes can be re-utilized for synthesis of NAD^+^ by the salvage cocktail, thus maintaining a typical host cell concentration of NAD^+^/NADH. This process apparently enabled NAD^+^/NADH dependent redox reactions without the need for addition of external NAD^+^ at high temperatures.

## Discussion

Enzymatic bioconversion has great potential for use in development of sustainable chemical-manufacturing processes [24]. In contrast to cell-based bioconversion, in vitro enzyme-catalyzed bioconversion allows separation of cell growth and enzymatic conversion phases. This phase separation allows optimization of each phase with less complexity [25, 26]. Enzymes from thermophiles have several advantages over mesophilic enzymes; their high thermal stability, for example, allows relatively easy purification. Nicotinamide cofactors, NAD^+^/NADH, which are indispensable for wide variety of enzymatic bioconversion, are, however, thermolabile, a property that limits use of thermophilic enzymes for oxidation–reduction reactions. This limitation is an important issue for in vitro metabolic engineering with thermostable enzymes at high temperatures. Thus, easy, simple, and user-friendly means to overcome this problem are needed.

In this study, an *E. coli* strain was engineered to produce an enzyme cocktail for NAD^+^ salvage synthesis that enabled sustainable bioconversion without external addition of nicotinamide cofactors. This integrated strain can provide a solution for maintaining a constant concentration of nicotinamide cofactor, NAD^+^/NADH, at high temperatures along with enzymes for ATP regeneration.

The salvage synthesis cocktail maintained NAD^+^ concentration at 1 mM for 12 h, but this concentration decreased thereafter (Fig. 6). Considering an NAD^+^ half-life of 6 h (Fig. 6), degradation rate of NAD^+^ at a concentration of 1 mM is 1.9 µM/min. The salvage cocktail was used at a concentration of 30 mg wet cells/mL, which was equivalent to NAD^+^ salvage synthesis capacity of 2.2 µM/min even after 24 h (calculated from Fig. 4), being sufficient for salvage of 1 mM of NAD^+^. This calculation indicates that the gradual decrease of NAD^+^ concentration was not due to thermal inactivation of thermophilic enzymes composing the cocktail.

During NAD^+^ salvage experiments with enzyme cocktail (Fig. 6), a sharp decrease in ATP concentration and a sharp increase in AMP concentration after 12 h (Additional file 1: Figure S5) was observed. Salvage synthesis of NAD^+^ converts 3 molecules of ATP to 3 molecules of AMP to produce each molecule of NAD^+^. This is corresponding to consumption of six high-energy phosphate bonds. The consumption rate of high-energy phosphate to maintain 1 mM NAD^+^ is therefore 0.68 mM/h. A shortage of ATP is likely a limiting factor for continuous salvage synthesis of NAD^+^ at high temperatures. ATP consumption was also detected in the negative control, which indicated degradation of ATP to ADP and AMP by residual host-derived enzymes and reverse reactions of PPK and ADK.

A continuous supply of ATP is critical for sustaining NAD^+^ salvage synthesis. In addition to the ATP regeneration system with polyphosphate, PPK and ADK, different processes such as glycolysis with different inorganic phosphate-dependent phosphorylation can be applied for effective regeneration [27].

In construction of the integrated strain, protein expression levels were modulated by arranging the order of genes in a single operon. As a result, the rate limiting enzyme, NADS, was encoded by the first gene in the operon (Fig. 3c). Expression of the first gene is dependent on the strength of a promoter; performance of the salvage cocktail can be improved by using stronger promoters. However, precise prediction of gene-expression levels is still challenging. Controlling the amount of mRNA solely by the order of genes in an operon is not perfect, resulting, in this case, in excessive expression of downstream genes (Fig. 3b). This leads to redundant production of some enzymes, and wasted energy in the integrated strain [28]. Random combination of variety promoters and/or ribosome binding sites as well as the order of genes will give us the best combination for gene expression, when a high-throughput screening assay for the ability of NAD^+^ salvage synthesis is established [29].

One of the advantages of using enzymes is their exchangeability. By screening or designing enzymes with better performance, the performance of in vitro pathway can be accumulatively improved. Salvage synthesis of NAD^+^ is widely distributed among thermophilic bacteria as well as hyperthermophilic archaea as a means to address thermal instability of NAD^+^ [30, 31]. A diverse compendium of enzymes is available for consideration for inclusion in a NAD^+^ salvage synthesis system. Continuous improvement of this system can be anticipated.

## Conclusions

We constructed a strain for salvage synthesis of NAD^+^ by co-expressing six thermophilic enzymes in a defined manner. The NAD^+^ salvage synthesis cocktail prepared from the constructed strain is capable of using thermal decomposition products to maintain 1 mM of NAD^+^ at 60 °C for 12 h and 0.1 mM of NAD^+^ for at least 28 h. Further, the enzyme cocktail enabled NAD^+^/NADH-dependent bioconversion without external addition of nicotinamide cofactors, maintaining the concentration of cell-derived NAD^+^/NADH through resynthesis of NAD^+^.

## Methods

### Bacterial strains, plasmids and oligonucleotides

Bacterial strains, plasmids and oligonucleotides used in this study are listed in Additional file 1: Table S1.

### Plasmid construction

Genes coding for NAMase, ADPRP, RPK, NaPRT, NaMAT and NADS were codon-optimized and synthesized along with the identical ribosome binding site (RBS) sequence by GeneArt Gene Synthesis (Thermo Fisher Scientific, USA). DNA of optimized genes with RBS was cloned into the plasmid, pRCI, through restriction digestion with BspQI, ligation and transformation into *E. coli* DH5α. The DNA sequence of plasmid was confirmed to be correct by sequencing (FASMAC, Japan). The complete sequences of codon-optimized genes are available in Additional file 1. A gene encoding PPK from *R. marinus* was amplified from the genomic DNA by PCR with respective primers, and cloned into *Nde*I-digested pET21a via In-Fusion^®^ HD Cloning Kit (Takara, Japan).

### Culture conditions and preparation of cell materials

For determination of enzyme activity and mRNA abundance in each strain, *E. coli* DH5α harboring pRCI was cultivated in Lysogeny Broth (LB) medium [32] supplemented with 100 µg/mL of ampicillin at 25 °C and 250 rpm until OD reached around 0.1, and further cultivated at 42 °C and 250 rpm for 1 h to induce protein production. For RNA isolation, 1 mL of cell culture was centrifuged at 16,000×*g* for 1 min, briefly washed once with 50 mM HEPES–NaOH (pH 8), immediately frozen in liquid nitrogen, and stored at − 80 °C until further use. For enzyme activity measurement, cell cultures were centrifuged at 3000×*g* for 10 min, washed twice with 50 mM of HEPES–NaOH (pH 8), and stored at − 20 °C until further use.

For preparing cell material for enzymatic conversion, *E. coli* DH5α with plasmid was cultivated in Terrific Broth (TB) medium supplemented with 10 mM of MgCl_2_ and 100 µg/mL of ampicillin [32, 33] at 25 °C and 250 rpm until OD reached approximately 4. Protein production was then induced by incubation at 42 °C and 250 rpm. *E. coli* Rosetta2(DE3)pLysS harboring pET21 was cultivated similarly with the exception that protein production was induced by addition of 1 mM IPTG and performed at 37 °C and 250 rpm. Cells were harvested by centrifugation and washed twice with 50 mM of HEPES–NaOH (pH 8). Cell pellets were stored at − 20 °C until further use.

### Preparation of thermophilic enzyme solutions

Cell pellets were resuspended at a concentration of 200 mg wet cells/mL in a resuspension buffer consisting of 400 mM HEPES–NaOH (pH 8) and 10 mM MgCl_2_. For preparation of thermophilic enzyme solutions, cells were disrupted by sonication, and the supernatant after centrifugation was subjected to heat treatment at 70 °C for 30 min. The soluble fraction after centrifugation was used as thermophilic enzyme solution.

### Quantification of enzyme activity with spectrophotometry

Enzyme activities were determined as previously described [18]. Briefly, substrate was continuously converted to NADH through a coupled reaction at 60 °C, and NADH concentration was measured by absorbance changes at 340 nm using a UV-2450 spectrophotometer (Shimazu, Japan) (Additional file 1: Figure S2). The standard mixture was composed of 400 mM HEPES–NaOH (pH 8), 60 mM NH_4_Cl, 10 mM MgCl_2_, 1 mM polyphosphate (Cat: 28-2880-5; Sigma, Germany), 0.2 mM ATP, 1 mM glucose and an excess amount of glucose dehydrogenase (GDH). A moderate amount of the enzyme of interest and an excess of enzymes catalyzing downstream reactions of NAD^+^ salvage synthesis was added to the standard mixture, so that NADH production rate reflected the activity of the enzyme of interest. Substrates were used at the final concentration of 0.2 mM for determining enzyme activity as follows; deamino-NAD^+^ (NaAD) for NADS, nicotinate mononucleotide (NaMN) for NaMAT, nicotinate (NA) and phosphoribosyl pyrophosphate (PRPP) for NaPRT, NA and ribose-5-phosphate (R5P) for RPK, NA and ADP-ribose for ADPRP, and NAM and PRPP for NAMase, respectively. The overall performance of the integrated strain was assessed using NAM and ADP-ribose as substrates. Reaction mixtures were incubated at 60 °C for 1 min prior to substrate addition.

### Quantification of mRNA expression level by qRT-PCR

Total RNA was isolated using RNeasy kits (Qiagen, Germany) according to the manufacture’s protocol, and quality and quantity was assessed by electrophoresis and Nanodrop (Thermo Fisher Scientific, USA). Equal amounts (ng) of isolated RNAs were used as starting materials for reverse transcription. Total RNA was converted to cDNA with ReverTra Ace (Toyobo, Japan), and RNA abundance was quantified with THUNDERBIRD SYBR qPCR mix (Toyobo, Japan) and StepOnePlus Real-Time PCR system (Applied Biosystems, USA) using appropriate primers (Additional file 1: Figure S1). Abundance of mRNA was normalized to OD_600_ of cell cultures used for RNA isolation, and normalized mRNA abundance was further compared across strains.

### Gene assembly with OGAB method and construction of integrated strain

Gene assembly was performed as previously described [20, 34] with minor modifications (Additional file 1: Figure S3). Briefly, pUC19 V-6th was constructed from pUC19V-1st by PCR with respective primers, restriction digestion by *Sal*I and transformation into *E. coli* DH5α. DNA of optimized genes was cloned into each pUC19 destination vector through restriction digestion with BspQI, ligation and transformation, resulting in pUC19V-1st-NADS, pUC19V-2nd-NaPRT, pUC19V-3rd-ADPRP, pUC19V-4th-NaMAT, pUC19V-5th-RPK, pUC19V-6th-NAMase. After confirmation of the DNA sequence, plasmids were digested with *Dra*III, resulting in DNA fragments containing optimized gene sequences and unique cohesive ends for ordered gene assembly. An *E. coli*–*B. subtilis* shuttle vector, pGETS118, was digested by *Sfi*I. Equimolar amounts of DNA fragments and digested pGETS118 were incubated at 37 °C for 30 min in the presence of T4 DNA ligase, 66 mM Tris–HCl (pH 7.6), 6.6 mM MgCl_2_, 10 mM dithiothreitol, 100 µM ATP, 150 mM NaCl and 10% PEG6000. The reactant was transformed into competent *B. subtilis* BUSY9797 cells, and the plasmid was extracted from a tetracycline resistant colony. The gene order was confirmed by PCR using respective primers and electrophoresis, and the resulting plasmid was named pGETS118-NAD^+^. An integrated strain was constructed by co-transformation of pGETS118-NAD^+^ and pBR-CI857 into *E. coli* DH5α, resulting in *E. coli* DH5α (pBR-CI857, pGETS118-NAD^+^).

### Screening of PPK

A reaction mixture was composed of 400 mM HEPES–NaOH (pH 8), 60 mM NH_4_Cl, 10 mM MgCl_2_, 1 mM ADP, 1 mM glucose, 10 mM polyphosphate, glucose kinase (GK), glucose-6-phosphate dehydrogenase (G6PDH), and heat-purified PPK. Polyphosphates from different sources were used as phosphate donors. GK was prepared from the strain harboring pET11-TTHA0299 (Q5SLJ4) [8], and G6PDH (code number, GLD-75-01) was purchased from Thermostable Enzyme Laboratory (Kobe, Japan). GK and G6PDH was added in an excess amount. The absorption of NADH at 340 nm was monitored, and the activity of PPK was calculated from changes of absorbance.

### Confirmation of NAD^+^ salvage synthesis capability of the integrated strain

The reaction mixture was composed of 400 mM HEPES–NaOH (pH 8), 60 mM NH_4_Cl, 10 mM MgCl_2_, 1 mM polyphosphate, 3 mM ATP and 0.1 or 1 mM NAD^+^. Enzyme solutions prepared from 200 mg wet cells/mL of each strain were added at the following ratio (vol/vol) to the total reaction volume; 15% for the NAD^+^ salvage cocktail, 2.5% for ADK and 5% for PPK. Enzyme reactions were performed at 60 °C.

### Quantification of NAD^+^, ATP, ADP, and AMP concentration by HPLC

Concentrations of NAD^+^, ATP, ADP and AMP were determined using a Prominence HPLC system (Shimazu, Japan). Samples were mixed with an equal volume of 0.4 M HCl to stop the enzymatic reaction, and centrifuged at 15,000×*g* for 10 min at 4 °C to remove debris. The supernatant was separated with a column, UK-C18, (Imtakt, Japan) at 40 °C with 5 g/L (NH_4_)_2_HPO_4_ (pH 2.8 adjusted with H_3_PO_4_) as the mobile phase at a flow rate of 1 mL/min. Peak intensities at 254 nm were compared to appropriate standards to assess concentrations of each compound in the sample.

### Enzymatic coupled reaction for lactate production with NAD^+^ salvage cocktail

The reaction mixture was composed of 400 mM HEPES–NaOH (pH 8), 60 mM NH_4_Cl, 10 mM MgCl_2_, 1 mM polyphosphate, 100 mM glucose, and 100 mM pyruvate. Enzyme solutions prepared from 200 mg wet cells/mL of each strain were added at the following ratio (vol/vol) to the total reaction volume; 15% for the NAD^+^ salvage cocktail, 2.5% for ADK, 5% for PPK, 5% for LDH and 5% GDH. Reaction was performed at 60 °C. As a negative control, an enzyme solution prepared from an *E. coli* strain expressing no thermophilic enzymes, *E. coli* DH5α (pBR-CI857, pGETS118), was used instead of the NAD^+^ salvage cocktail.

### Quantification of lactate production and glucose consumption by biosensor

Concentrations of lactate and glucose in a sample were measured with biosensor BF-5 and corresponding enzyme electrodes connected in series (Oji Scientific Instruments, Japan) at 30 °C. The reactant was appropriately diluted before measurement, and signal intensities were compared to standards with known concentrations.

### Quantification of NAD^+^/NADH concentration by fluorescent assay

Total amounts of NAD^+^ and NADH were measured using a fluorescence-based cycling assay. The assay mixture consisted of 100 mM HEPES–NaOH (pH 8), 0.5 mM phenazine methosulfate, 0.4 mM resazurin, 10 mM of absolute ethanol and 5 U/mL alcohol dehydrogenase from yeast (Oriental Yeast, Japan). Appropriately diluted samples (10–40 µL) were mixed with assay mixture, and changes in fluorescence intensity over time were monitored with a microtiter plate and plate reader, GENios, (Tecan, Switzerland) at 37 °C (excitation wavelength, 540 nm; emission wavelength, 586 nm). Total concentration of NAD^+^ and NADH was quantified in comparison to a standard with known concentrations.

## Additional file

**Additional file 1.** Additional figures and table.

## Abbreviations

ADK: adenylate kinase
ADPRP: ADP-ribose pyrophosphatase
GDH: glucose dehydrogenase
LDH: lactate dehydrogenase
NA: nicotinate
NaAD: deamino-NAD^+^
NADS: NAD^+^ synthase
NAM: nicotinamide
NAMase: nicotinamidase
NaMAT: nicotinate mononucleotide adenylyltransferase
NaMN: nicotinate mononucleotide
NaPRT: nicotinate phosphoribosyltransferase
OGAB: ordered gene assembly in *Bacillus subtilis*
PPK: polyphosphate kinase
PRPP: phosphoribosyl pyrophosphate
R5P: ribose-5-phosphate
RPK: ribose-phosphate pyrophosphokinase
