# Supplementary material for: Developing a single strain for in vitro salvage synthesis of NAD+ at high temperatures and its potential for bioconversion
Source: Microb Cell Fact. 2019 Apr 25;18:75. doi: 10.1186/s12934-019-1125-x (PMC6482498; doi:10.1186/s12934-019-1125-x)
Supplement: Supplementary file 1 — Additional file 1. Additional figures and table. [file 12934_2019_1125_MOESM1_ESM.docx]

## Additional file to

### Title

### Developing a single strain for *in vitro* salvage synthesis of NAD^+^ at high temperatures and its potential for bioconversion

### Corresponding author

^1^Kohsuke Honda

### Other authors

^1^Hironori Taniguchi, ^2^Makoto Imura, ^1^Kenji Okano, ^1^Kohsuke Honda

^1^Department of Biotechnology, Graduate School of Engineering, Osaka University

Yamadaoka 2-1, Suita, Osaka, 565-0871, Japan

^2^KOHJIN Life Sciences Co., Ltd.

Higashihama 1-6, Saiki, Oita, 876-858-, Japan

### Content

**The complete sequences of codon optimized genes**

**Table S1 Bacterial strains, plasmids and oligonucleotides used in this study**

**Figure S1 Primer location for qRT-PCR**

**Figure S2 Measurement of enzyme activities with spectrophotometry**

**Figure S3 OGAB method scheme**

**Figure S4. NAD^+^/NADH dependent redox reactions with 0.1 mM of NAD^+^**

**Figure S5. ATP, ADP and AMP concentration during salvage synthesis of 1 mM NAD^+^**

#### **The complete sequence of codon optimized genes**

**>NADS**

TAGGCTCTTCAGTGAAGAAGGAGATATACAT**ATG**CAAGAAAAAATTGATAAACTGGTGCAGTGGCTGCGTGATCAGGTTAGCAGCGCAGGTCTGAATGGTGCAGTTGTTGGTATTAGCGGTGGTATTGATAGCGCAGTTGTTGCACATCTGATTAAACGTGCATTTCCGGATGATAGCCTGGGTCTGATTATGCCGTGTAAAAGCAATCCGAAAGATATGGAAGATGCCCTGAAAGTTGTTAAAAGCTGTGGTATTCGTCATCTGGTTATTGATCTGACCGAAGCACATCGTACCCTGTTTGGTGCAGTTGAAGCAGAACTGAAAGCAATTGGTGAATGGTCTGAAGAACGTGCACGTCTGGGTGATGCAAATACCCGTGCCCGTCTGCGTATGACCACCCTGTATGCAGTTGCAAATAACTATGGTTATCTGGTTGTTGGCACCGATAATGCAGCAGAATGGCATACCGGTTATTTCACCAAATATGGTGATGGTGGTGTTGATCTGGTTCCGCTGATTCACTTTACCAAAGGTGAAGTTCGTGAAATGGGTCGTCTGCTGGGTGTTCCGGAAGAAATTATCAAAAAAGCACCGTCAGCAGGTCTGTGGGAAGGTCAGACCGATGAAAGCGAAATGGGCACCACCTATGAAATGATCGATAAATATCTGAAAGGCGAAGAAATTCCGGAACGTGATCGTAAAATCATTGAACGTCTGCATGAACGCAGCCATCATAAACGTCAGCTGGCAATTGCACCGCCTAAATTT**TGA**TAATGAAGAGCCTA

**>NaPRT**

TAGGCTCTTCAGTGAAGAAGGAGATATACAT**ATG**GATCCGTTTGGCATTCTGTATACCGATCTGTATCAGCTGACCATGGGTCAGGTTTATTTTCGTATGGGTCTGCATGAAAAAGAAGCACTGTTTGAAGCATTTTATCGTCGCAATCCGGATTATGGTGCACATCAGGCAGGTTATACCGTTTTTGCAGGTCTGGATCCGCTGCTGAGCTGGATGGAAGAAGCACGTTTTGGCGAAGAAGAAATTGCAGCACTGCGTAATCTGAAAAGCCGTAGCGGTAAACCGCTGTTTGCAGAAGATTATCTGCGTTATCTGAAAGCCATGGGTGGTTTTAGCGGTCTGACCCTGCGTGCACTGCCGGAAGGTCGTGTTGCACATCCGCAGGTTCCGCTGGTTAGCGTTGAAGGTCCGCTGCTGCAGGCACAGCTGCTGGAAACCGCACTGCTGAATCGTCTGAATTATGAAACCCTGATTGCAACCAAAGCAAGCCGTGTTCGTGAAGCAGCCGGTGAAGCAGTTGTTCTGGAATTTGGTCTGCGTCGTGCACCGGCAAAAGGTGGTGAAAGCGCAACCCGTGCAAGCCTGATTGGTGGTGCAAATCGTAGCAGCGCAGTTAGCCTGAGCCATCTGCTGGGTCTGCCTGCAAGCGGCACCCATGCACATAGCCTGGTTCAGGCATTTATGGCACTGGGTTATAGCGAAGAAGATGCATTTCGCGCATTTGCCGAAGTTTTTCCGGATGATGCAATTCTGCTGCTGGATACCGTTGATACCCTGCATAGCGGTCTGCCGCATGCAATTCGTGTTTTTGAAGAACTGCGTCGTAAAGGTCATCGTCCGGTTGGTGTTCGTATTGATAGCGGTGATCTGGCACATCTGGCAATTCAGGTTGCACGTGAACTGGATCGTGCAGGTTTTCCGGAAGTTCTGATTGTTCTGAGTGGTGATCTGGATGAACTGGTTATTTGGCAGATTAAAAGCCAGATTCTGGAAGAGGCACCGCGTTATGGTGTTGATCCGGATCGTCTGCTGAAACGTCTGGTTTATGGTGTTGGCACCCGTATGGTTGTTAGCTGGGGTGCCCCTGCCCTGGGTGGTGTTTATAAACTGGTTGCAGTTCGTGAAAATGGCACCTGGGCACCGGCAATGAAAGTTAGCAATAGCCTGGAAAAAGTTCTGAATCCGGGTCATAAACGTGTTTATCGTGTGTATGATGAACGTGGTCTGGCAACCGCAGATCTGCTGGCACTGGCCGAAGAAGAAGTTCGCGAGGACGCACCGCTGACCCTGCGCCATCCGACCGATCCGACCAAACGTCGTACCCTGCGTCCGGGTAGCTTTCGTCTGGAACCTCTGCTGGAAGAAGTTTATCGCGGTCGTCGTCTGTTTCCGCCTCTGCCGCTGGAAGAACTGCGCAAACGTCGTGATCGTGATGTTGCACTGCTGGATCCGGGTGTTCGTCGTCTGGTTAATCCGCATGTTTATCATGTTAGCCTGACCGAACGTCTGTTTGCACTGAAAGAAGAACTGGTTCGTCGCCTGGAAGGT**TAA**TAATGAAGAGCCTA

**>NaMAT**

TAGGCTCTTCAGTGAAGAAGGAGATATACAT**ATG**CGCATTGGTCTGTTTGGTGGTAGCTTTGATCCGATTCATCTGGGTCATCTGCTGGCAGCAAGCCAGGCACAAGAGGTTCTGTGTCTGGATCGTGTTCTGTTTGTTGTTGCAGCACGTCCGCCTCATAAAGTTCCGGTTGCCCCTGCCGAAGCACGTTATGAAATGACCCTGCTGGCCGTTGCAGAAGATCCGCGTTTTACCGTTAGCCGTCTGGAACTGGATCGTCCGGGTCCGAGCTATACCGTTGATACCCTGCGTGAAGCACGTCGTCTGTTTCCGCAGGATGAACTGTTTTTTATCACCGGTGCAGATGCATATCGTGATGTTCTGACCTGGAAAGAAGGTGAACGTCTGCCGGAATATGCAACCCTGGTTGCAGTTGCACGTCCGGGTTATCCGCTGGAAGAAGCACCGCTGCCGGTTGTTCCGCTGTTTGTTCCGGAAGTTGGTATTAGCAGCACCGAAATTCGTCGTCGTCTGAAAGAAGGCCGTAGCGTTCGTTATTGGGTTCCGCGTGCAGTTGAAGTGTATATTGAAAAACATGGCCTGTACCGC**TAA**TAATGAAGAGCCTA

**>NAMase**

TAGGCTCTTCAGTGAAGAAGGAGATATACAT**ATG**AAACCGGCACTGGTTGTTGTGGATATGGTGAACGAATTTATTCATGGTCGTCTGGCAACACCGGAAGCAATGAAAACCGTTGGTCCGGCACGTAAAGTTATTGAAACCTTTCGTCGTAGCGGTCTGCCGGTTGTTTATGTTAATGATAGCCATTATCCGGATGATCCGGAAATTCGTATTTGGGGTCGTCATAGCATGAAAGGTGATGATGGTAGCGAAGTGATTGATGAAATTCGTCCGAGTGCCGGTGATTATGTTCTGGAAAAACATGCATATAGCGGCTTTTATGGCACCAACCTGGATATGATTCTGCGTGCAAATGGTATTGATACCGTTGTTCTGATTGGTCTGGATGCCGATATTTGTGTTCGTCATACCGCAGCAGATGCACTGTATCGTAATTATCGTATTATCGTTGTTGAAGATGCAGTGGCAGCACGTATTGATCCGAATTGGAAAGATTATTTCACCCGTGTTTATGGTGCAACCGTTAAACGTAGTGATGAAATTGAAGGTATGCTGCAAGAAGATCAGATCGAAACC**TAA**TAATGAAGAGCCTA

**>RPK**

TAGGCTCTTCAGTGAAGAAGGAGATATACAT**ATG**GAAATCCGTCTGTTTAGCGGTAGCGCACATCCGGATCTGGCACGTCGCGTTGCCGAAGCCCTGGGTGTTCCGCTGGGTAAAGCACTGGTTGATCGTTTTCCGGATGGTGAAGTTCGTGTTCGTCTGCTGGAAAGCGTTCGTGGTGAAGATGTTTATCTGATTCAGCCGACCTGTCCGCCTGTTAATGATCATCTGATGGAACTGCTGCTGCTGGCAGATGCAGCACGTCGTAGCAGCGCAGGTCGTATTAATGCAGTTATTCCGTATTTTGGTTATGCCCGTCAGGATAAACAGACCGAAGGTCGTGAACCGATTAGCGCACGTCTGGTTGCAGGTCTGCTGGAACGTGTTGGTGTTGAACGTGTTATTGCAATTGATCTGCATGCACCGCAGATTCAGGGTTTTTTTGATATTCCGGTTGATCATCTGAGCGCAGTTCGTCTGTTTGCACGTTATCTGCGTGAAAAAGGTTATGTTGAAAATGCCGTTGTTGTTAGTCCGGATGCAGGTCGTGCAGAAGAAGCACGTCGTCTGGCAGAACGTCTGGGTCTGCCGCTGGCAATGCTGGCAAAACGTCGTCTGGGTCCGCGTGAAACCAGCGTTACCTATGTTATTGGTGATGTTGAAGGTAAACGTCCGATTCTGGTTGATGATATTGTTAGCACCGGTGGCACCATTCGTCGTGGTGTTGAAGCACTGCTGCAGGCAGGCGCACTGCCGGAAGTTGTTGTTATGGCAACCCATGCAGTTCTGGTTGGTGGTGCAAAAGAAAATCTGGCACATCCGGCAGTTCGTGAAGTTGTTTTTACCGATACCATTCCGCTGAAAGATGGTGGTTATACCGTTCTGAGCACCGCAGAACTGCTGGCACAGGCAATTCGTCATGTTCATACCAATCAGAGCGTTAGCGCACTGATC**TAA**TAATGAAGAGCCTA

**>ADPRP**

TAGGCTCTTCAGTGAAGAAGGAGATATACAT**ATG**GGTCGCGTTTATTATGGTGGTGTTGAACGTACCTATCTGTATCGTGGTCGTATTCTGAATCTGGCACTGGAAGGTCGTTATGAAATTGTTGAACACAAACCGGCAGTTGCAGTTATTGCCCTGCGTGAAGGTCGTATGCTGTTTGTTCGTCAGATGCGTCCGGCAGTTGGTCTGGCACCGCTGGAAATTCCGGCAGGTCTGATTGAACCGGGTGAAGATCCGCTGGAAGCAGCCCGTCGTGAACTGGCAGAAGAAACCGGTCTGAGCGGTGATCTGACCTACCTGTTTAGCTATTTTGTTAGTCCGGGTTTCACCGATGAAAAAACCCATGTTTTTCTGGCCGAAAACCTGAAAGAAGTTGAAGCACATCCGGATGAAGATGAAGCAATTGAAGTTGTTTGGATGCGTCCGGAAGAAGCACTGGAACGTCATCAGCGTGGTGAAGTTGAATTTAGCGCAACCGGTCTGGTTGGTGTTCTGTATTATCATGCATTTCTGCGTGGTCGC**TAA**TAATGAAGAGCCTA

* Red characters indicate the recognition site of BspQI. Bold characters with underline and bold characters indicate initiation codons and stop codons of each gene, respectively.

#### Table S1. Bacterial strains, plasmids and oligonucleotides used in this study

| **Bacterial strain** | **Discription** | **Reference** |
| --- | --- | --- |
| ***Escherichia coli*** |  |  |
| DH5α | F^−^ ϕ80d*lacZ*ΔM15 Δ(*lacZYA-argF*)U169 *deoR* *recA*1 *endA*1 *hsdR*17(r_K_^−^ m_K_^+^) *phoA* *supE*44 *λ*^−^ *thi*-1 *gyrA*96 *relA*1 | [1] |
| Resetta2(DE3)pLys | F^-^ *ompT hsdS*_B_(r_B_^-^ m_B_^-^) *gal* *dcm* (DE3) pLysSRARE2 (Cm^R^) | Novagen |
| ***Bacillus subtilis*** |  |  |
| BUSY9797 | RM125 *proB*::(*cI-spc*) | [2] |
| **Plasmid** | **Discription** | **Reference** |
| pRCI | Expression vector with temperature inducible promoter Pr and *cI857* repressor. Ap^R^ | [3] |
| pRCI-NADS | pRCI based plasmid encoding NAD^+^ synthase from *Bacillus stearothermophilus* (NADS; A0A0K9HRP4^1^, codon optimized) | In this study |
| pRCI-NaMAT | pRCI based plasmid encoding nicotinate mononucleotide adenylyltransferase from *T. thermophilus* (NaMAT; Q5SHF0^1^, codon optimized) | In this study |
| pRCI-NaPRT | pRCI based plasmid encoding nicotinate phosphoribosyltransferase from *T. thermophilus* (NaPRT; Q5SKM6^1^, codon optimized) | In this study |
| pRCI-NAMase | pRCI based plasmid encoding nicotinamidase from *Thermoplasma acidophilum* (NAMase; Q9HKY9^1^, codon optimized) | In this study |
| pRCI-RPK | pRCI based plasmid encoding ribose-phosphate pyrophosphokinase from *T. thermophilus* (RPK; Q5SI31^1^, codon optimized) | In this study |
| pRCI-ADPRP | pRCI based plasmid encoding ADP-ribose pyrophosphatase from *T. thermophilus* (ADPRP; Q84CU3^1^, codon optimized) | In this study |
| pUC19V-1st-DraIII | pUC19 based plamid with unique DraIII restriction sites | [2] |
| pUC19V-2nd | pUC19 based plasmid with unique DraIII restriction sites | [2] |
| pUC19V-3rd | pUC19 based plasmid with unique DraIII restriction sites | [2] |
| pUC19V-4th | pUC19 based plasmid with unique DraIII restriction sites | [2] |
| pUC19V-5th | pUC19 based plasmid with unique DraIII restriction sites | [2] |
| pUC19V-6th-DraIII | pUC19 based plasmid with unique DraIII restriction sites | In this study |
| pUC19V-1st-NADS | pUC19 based plasmid with unique DraIII restriction sites encoding NADS (codon optimized) | In this study |
| pUC19V-2nd-NaPRT | pUC19 based plasmid with unique DraIII restriction sites encoding NaPRT (codon optimized) | In this study |
| pUC19V-3rd-ADPRP | pUC19 based plasmid with unique DraIII restriction sites encoding ADPRP (codon optimized) | In this study |
| pUC19V-4th-NaMAT | pUC19 based plasmid with unique DraIII restriction sites encoding NaMAT (codon optimized) | In this study |
| pUC19V-5th-RPK | pUC19 based plasmid with unique DraIII restriction sites encoding RPK (codon optimized) | In this study |
| pUC19V-6th-NAMase | pUC19 based plasmid with unique DraIII restriction sites encoding NAMase (codon optimized) | In this study |
| pBR-CI857 | pBR322 based plasmid with *cI857* repressor, Ap^R^ | [3] |
| pGETS118 | *E. coli*-*B. subtilis* shuttle vector, Pr, Tc^R^ | [4] |
| pGETS118-NAD^+^ | pGETS118 based vector encoding six genes necessary for NAD^+^ salvage synthesis | In this study |
| pET21-PPK_Tt_ | pET21 based plasmid encoding for polyphosphate kinase from *T. thermophilus* HB27 (PPK; Q72JY1^1^) | [5] |
| pET21-PPK_Rm_ | pET21 based plasmid encoding for polyphosphate kinase from *Rhodothermus marinus* (PPK; D0MHE7^1^) | In this study |
| pET11-ADK | pET11 based plasmid encoding for adenylate kinase from *T. thermophilus* (ADK; Q5SHQ9^1^) | RIKEN *T. thermophilus* HB8 expression plasmid library  [6] |
| pRCI-GDH | pRCI based plasmid encoding glucose dehydrogenase from *Sulfolobus solfataricus* (GDH; Q7LYI9^1^) | [7] |
| pET11-LDH | pET11 based plasmid encoding lactate dehydrogenase from *T. thermophilus* (LDH; Q5SJA1^1^) | [6] |
| **Oligo nucleotide** | **Sequence** | **description** |
| No.1 | TAGCGGCTTTTATGGCACCAAC | qPCR_pRCI-NADase_fwd |
| No.2 | CATCTGCTGCGGTATGACGAAC | qPCR_pRCI-NADase_rev |
| No.3 | GCTGGATGGAAGAAGCACGTTT | qPCR_pRCI-NaPRT_fwd |
| No.4 | TCAGACCGCTAAAACCACCCAT | qPCR_pRCI-NaPRT_rev |
| No.5 | TCTGTTTGTTGTTGCAGCACGT | qPCR_pRCI-NaMAT_fwd |
| No.6 | CGATCCAGTTCCAGACGGCTAA | qPCR_pRCI-NaMAT_rev |
| No.7 | CGTGCATTTCCGGATGATAGCC | qPCR_pRCI-NADS_fwd |
| No.8 | CACCAAACAGGGTACGATGTGC | qPCR_pRCI-NADS_rev |
| No.9 | GTTATTGCCCTGCGTGAAGGTC | qPCR_pRCI-ADPRP_fwd |
| No.10 | CCGGTTTCTTCTGCCAGTTCAC | qPCR_pRCI-ADPRP_rev |
| No.11 | GAAGTTCGTGTTCGTCTGCTGG | qPCR_pRCI-RPK_fwd |
| No.12 | GCAGCAGCAGTTCCATCAGATG | qPCR_pRCI-RPK_rev |
| No.13 | CTTTAAGAAGGAGATATACATATGGCCCGCACGGCAACA | *R._marinus*_PPK_fwd_pET21 |
| No.14 | TCCACCAGTCATGCTAGCCATCAGATCGGCCTGTGGTCC | *R._marinus*_PPK_rev_pET21 |
| No.15 | AACGAGCTCTTCTCACCATGTGAATTCAC | pUC19-6th-SacI_R |
| No.16 | AACGAGCTCTTCCTAACACTCTGTGGCTTCCTC | pUC19-stop-DraIII |

^1^ Accession numbers of UniProtKB are shown.


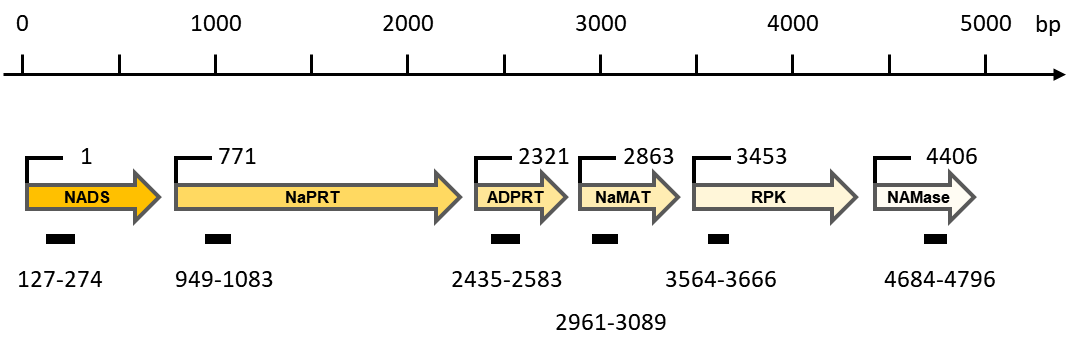


#### **Figure S1. Primer location for qRT-PCR**

Arrows represent each gene and the numbers above indicates the location of the first nucleotide in each gene. Black bars below arrows represent regions of amplicon during qRT-PCR, and the numbers below indicates the first and last nucleotide in each amplicon. The primer sequences are shown in Table S1. Primer construction for qRT-PCR was designed using Primer3. Upon selection of primers, the size of products was set between 100 and 150 bp, and the position of forward primers was set between 75 and 300 nucleotides from the initiation codon of each gene. Linearity of amplification of each gene with corresponding primer pair was confirmed using a corresponding plasmid DNA encoding a single gene as a template.


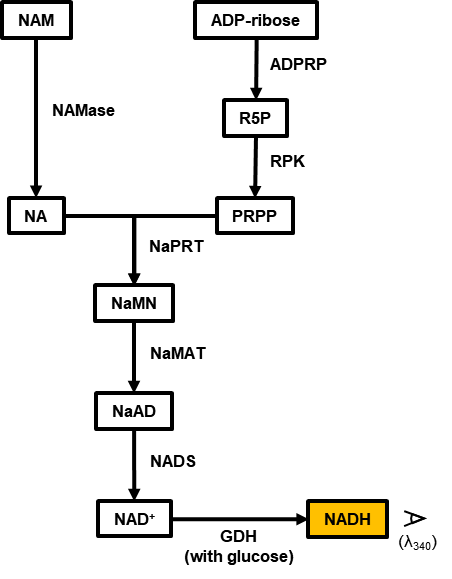


#### **Figure S2. Measurement of enzyme activities with spectrophotometry**

Quantification of enzyme activity with spectrometer is illustrated. Different substrates were used as a starting material depending on the targeted enzyme. Enzymes catalyzing the latter reaction were added in an excess amount so that the targeted reaction was the rate limiting reaction. NAD^+^ synthesized though NAD^+^ salvage synthesis enzymes were continuously converted to NADH by excess amount of GDH. The absorption of NADH at 340 nm was monitored, and the activity of the targeted enzyme was calculated from the change of the absorbance.


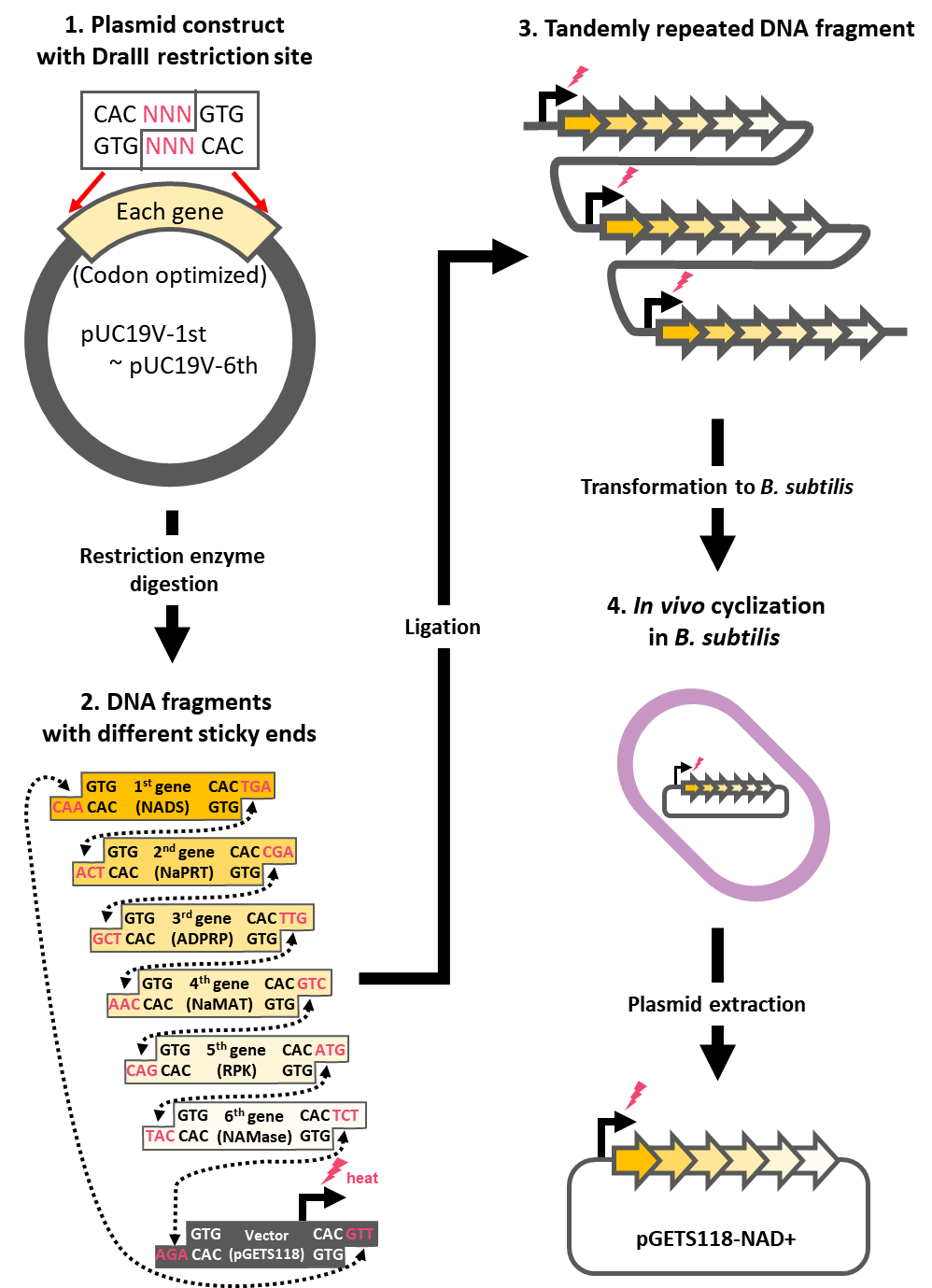


#### **Figure S3. OGAB method scheme**

A scheme of OGAB method is illustrated. Different sticky end sequences of pUC19V-1st~pUC19V-6th are also shown in the figure.


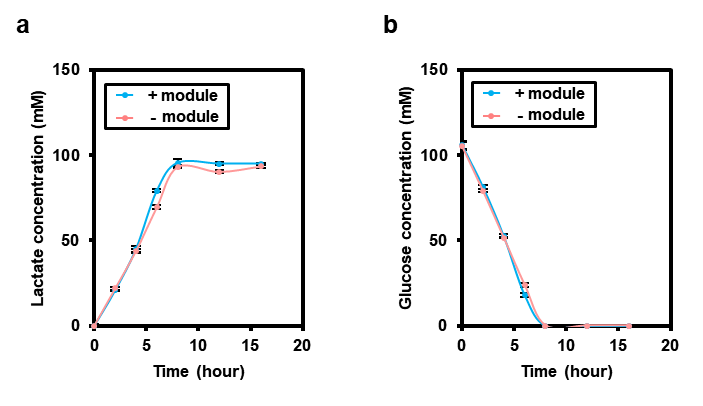


#### **Figure S4. NAD^+^/NADH dependent redox reaction with 0.1 mM of NAD^+^**

Lactate (a) and glucose (b) concentration were measured every 8 hours for 40 hours with the enzyme cocktail (+ cocktail) or in the negative control (- cocktail). NAD^+^ was supplemented at a concentration of 0.1 mM. Error bars represent standard errors calculated from triplicate measurements.


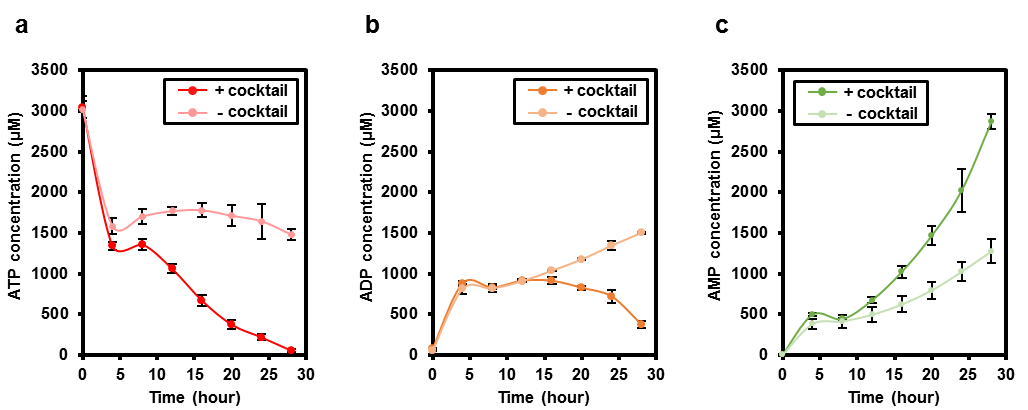


#### **Figure S5. ATP, ADP and AMP concentration during the salvage synthesis of 1 mM NAD^+^**

Concentrations of ATP (a), ADP (b) and AMP (c) are shown. Red, orange, and green line indicate ATP, ADP and AMP concentration with the enzyme cocktail (+ cocktail), respectively. Pale red, pale orange, and pale green line indicate ATP, ADP and AMP concentration in the negative control (- cocktail), respectively. Quantification was done by HPLC described in the methods section. Error bars represent standard errors calculated from triplicate measurements.

## References

1. Taylor RG, Walker DC, McInnes RR. *E. coli* host strains significantly affect the quality of small scale plasmid DNA preparations used for sequencing. Nucleic Acids Res. 1993;21:1677–8.

2. Hiroe A, Tsuge K, Nomura CT, Itaya M, Tsuge T. Rearrangement of gene order in the *phaCAB* operon leads to effective production of ultrahigh-molecular-weight poly[(R)-3-hydroxybutyrate] in genetically engineered *Escherichia coli*. Appl Environ Microbiol. 2012;78:3177–84.

3. Ninh PH, Honda K, Sakai T, Okano K, Ohtake H. Assembly and multiple gene expression of thermophilic enzymes in *Escherichia coli* for *in vitro* metabolic engineering. Biotechnol Bioeng. 2015;112:189–96.

4. Kaneko S, Akioka M, Tsuge K, Itaya M. DNA shuttling between plasmid vectors and a genome vector: systematic conversion and preservation of DNA libraries using the *Bacillus subtilis* genome (BGM) vector. J Mol Biol. 2005;349:1036–44.

5. Honda K, Hara N, Cheng M, Nakamura A, Mandai K, Okano K, et al. *In vitro* metabolic engineering for the salvage synthesis of NAD^+^. Metab Eng. 2016;35:114–20.

6. Yokoyama S, Hirota H, Kigawa T, Yabuki T, Shirouzu M, Terada T, et al. Structural genomics projects in Japan. Nat Struct Mol Biol. 2000;7:943–5.

7. Honda K, Inoue M, Ono T, Okano K, Dekishima Y, Kawabata H. Improvement of operational stability of *Ogataea minuta* carbonyl reductase for chiral alcohol production. J Biosci Bioeng. 2017;123:673–8.
